# Acute Compartment Syndrome: A Short Narrative Review of the Risk Factors, Complications, and Medicolegal Impact of a Missed Diagnosis

**DOI:** 10.7759/cureus.97781

**Published:** 2025-11-25

**Authors:** Ruqaiya Al-habsi

**Affiliations:** 1 Surgery, The Royal London Hospital, London, GBR

**Keywords:** acute compartment syndrome (acs), delayed fasciotomy, fasciotomy, medical litigation, missed compartment syndrome

## Abstract

Acute compartment syndrome (ACS) is a frequently encountered surgical emergency in trauma and orthopaedics. Timely diagnosis and prompt surgical fasciotomy of the involved compartments prevent complications, which can cause long-term morbidity, rehabilitation, renal failure, multiorgan failure, and even death if management is delayed or diagnosis is missed. The aim of this review was to identify and summarise existing literature on ACS, its complications, and the medicolegal impact when a diagnosis is delayed or missed. A narrative review was conducted using evidence from EMBASE, PubMed, and Google Scholar. There is extensive literature on the diagnosis and presenting signs and symptoms of ACS. However, not the same can be said about the effect of ACS on kidney function, rhabdomyolysis, multiorgan failure, and death. ACS is associated with a multitude of complications and has systemic effects, including electrolyte imbalance, rhabdomyolysis, and renal failure. Overall, ACS is one of the most common causes of litigation in trauma and orthopaedics, and care must be taken to remember that not all ACS is associated with trauma and fractures, resulting in higher rates of delayed diagnosis. To avoid litigation and morbidity to patients, timely diagnosis of ACS is essential, combined with avoiding delays to fasciotomy. Overall, ACS can cause devastating effects to patients, even without a missed diagnosis, as its complications can range from toe clawing and Volkmann contractures to muscle necrosis, which develops into sepsis and can cause multiorgan failure and death.

## Introduction and background

Definition

Acute compartment syndrome (ACS) is a relatively frequent orthopaedic surgical emergency, especially in patients with long bone fractures such as the tibia. It can be defined as a rise in myofascial intracompartmental pressure (ICP), leading to impairment of tissue perfusion [1]. ACS can also be defined as a rise in ICP to more than 30 mmHg, which is the usual threshold used to aid diagnosis [2].

Epidemiology and causes

ACS is widespread, and some studies report an incidence of 3.1 per 100,000 individuals. In men, it is 10 times more likely to occur than in women; however, this can be attributed to the fact that compartment syndrome is most often related to fractures, which are more common in males than in females, with a ratio of 13:1 [3]. Some studies report an incidence of 7.3 per 100,000 in males, reflecting a strong association with high-energy trauma and tibia fractures [4]. Population-based studies continue to show obvious male predominance, often accounting for 70-80% of cases, more commonly in males aged 15-35 years, who are most exposed to high-energy mechanisms such as road traffic injuries and sports-related trauma. Tibial shaft fractures remain the single most common precipitating injury, with reported ACS rates ranging between 1% and 11%, depending on fracture pattern, soft-tissue disruption, and mechanism of injury [1,4].

Lower limb fractures, most commonly tibia fractures, can result in continuous haemorrhage and oedema, making the affected compartments at a higher risk for ACS development. In the military, prophylactic fasciotomy has become the standard, especially as they are more likely to have fractures as a result of high-energy trauma [4].

Internationally, the incidence figures vary. In the United States, large trauma registry analyses show ACS occurring in approximately 2-9% of all tibial shaft fractures [1]. In Europe, the reports match this, as seen in Scandinavian and UK trauma centres reporting a 3-8% incidence [4]. In Southeast Asia and China, reports are comparable to this, especially with the high incidence of road traffic accidents in urban areas. In major trauma centres or orthoplastic centres that commonly manage Gustilo-Anderson IIIB injuries, the rates of ACS are understandably higher, reflecting both increased ICP and soft-tissue compromise [4]. 

Aya et al. [1] identified key predictors for missed ACS, especially in polytrauma patients and high-incidence trauma networks, further highlighting systemic patterns that can result in diagnostic delays [1].

Diagnostic challenge

ACS is usually a clinical diagnosis. There are six main signs identified clinically that point to the diagnosis of ACS, known as the six P's: pain, poikilothermia, pallor, paraesthesia, pulselessness, and paralysis. The latter three are often a late sign diagnosis [3]. The pain associated with ACS is often out of proportion to the injury and persists despite adequate analgesia.

The Royal College of Nursing in the United Kingdom uses an observational scoring chart to monitor patients at risk for compartment syndrome. Patients at risk included in the observational chart include those with tibia, forearm, or high-energy distal radius fractures; patients with orthopaedic injury or intervention in addition to known coagulopathies or those on anticoagulants; patients with crush injuries; and those admitted due to high-energy trauma, including open fractures [5].

The chart includes a variety of scorable sections, including pain at rest, on passive movement, or pain since last analgesia (scores 0 if none, 1 if mild, 2 if moderate, and 3 if severe).

Unfortunately, there is a challenge with diagnosing unconscious patients and patients who received nerve blocks for their operations; therefore, an ICP device is often used for such patients. A total pain score of 5 or more and an individual pain parameter score of 3 or a clinical concern are escalated immediately to the responsible clinician as per local hospital guidelines [5].

The British Orthopaedic Association recommends that patients presenting with ACS be managed in accordance with the BOAST (British Orthopaedic Association Standards for Trauma) guidelines. This excludes any patients with chronic (exertional) compartment syndrome.

The BOAST guidelines for the diagnosis and management of compartment syndrome of the extremities provide clear guidance on both the assessment and appropriate surgical management of ACS. Some of these recommendations include the need to ensure that ACS assessment is routinely done and documented in patients with limb injuries, post-extremity surgery patients, and any patients who underwent prolonged procedures. Once patients are identified to have clinical findings suggestive of ACS, their circumferential dressings should be released to expose the skin, the limb elevated, and adequate analgesia given. All patients should be re-assessed in 30 minutes to determine whether the above interventions reduced the pain or not. Surgery involves decompression of all involved compartments [6].

Consequences of delays

The majority of ACS cases are detected in a timely manner, and complications are avoided. Unfortunately, in a portion of patients, a diagnosis of ACS can be missed, resulting in lifelong effects. Within three hours of impaired local tissue perfusion, local tissue ischaemia and tissue necrosis can start. Within five hours of ischaemia, muscle tissue necrosis occurs in the majority of patients. Unfortunately, if an ACS diagnosis remains missed, the necrosis can turn into joint contractures and limb-threatening infection, which can then result in limb amputation [1].

Literature gap and aims

There is insufficient published literature on the specificity of clinical signs for diagnosing compartment syndrome. In addition to this, the majority of publications focus on ACS related to trauma; unfortunately, many patients present without trauma, and this can often lead to a missed diagnosis. Most published literature consists of case reviews on the management or post-management complications of ACS.

This review aims to look at the risk factors of compartment syndrome (for both trauma- and non-trauma-related cases) and the complications of compartment syndrome stated in the literature to date, as well as the medicolegal impact that missed diagnosis or delayed management results in.

Accordingly, this review summarises current evidence on the clinical, diagnostic, and medicolegal aspects of ACS and provides actionable recommendations, including routine neurovascular assessment and documentation, improved multidisciplinary team education, and the adoption of standardised compartment-pressure local hospital protocols.

## Review

Methods

This is a narrative review of missed ACS, its risk factors, complications, medicolegal effects on both the orthopaedic surgical community and patients, and its impact and costs on the hospital and the government. As this is a narrative review rather than a systematic review, the PRISMA (Preferred Reporting Items for Systematic Reviews and Meta-Analyses) methodology was not applied. However, to ensure transparency and replicability, the databases searched included PubMed, EMBASE, and Google Scholar; the specific search terms used and inclusion/exclusion criteria have been clearly described below. The literature search was conducted between 01 May 2025 and 10 October 2025. Studies were reviewed for relevance based on title and abstract, with full-text assessment where necessary. No formal risk-of-bias assessment was performed, as this is not required for narrative reviews, but preference was given to recent human studies and clinically applicable evidence.

Search Terms and Inclusion Criteria

The search terms used to identify relevant papers included "compartment syndrome", "ACS", "acute compartment syndrome", "missed ACS", "ACS management delays", "delayed fasciotomy", "Volkmann contractures", "ACS risk factors", "Compartment medicolegal", and "compartment syndrome litigation".

The study inclusion period was not limited to a specific time frame, but more recently published papers were prioritised. An additional paper was opted in, published in 1881 by Richard von Volkmann and entitled "Ischaemic muscle paralysis and contractures," accessed from the PubMed database. All papers included human studies and all age groups and genders.

Exclusion Criteria

All animal studies were excluded. Additionally, compartment syndrome not involving the upper or lower limbs was not included, as this paper primarily explored ACS from a trauma and orthopaedics surgical perspective. Papers on chronic (exertional) compartment syndrome were excluded from this literature review.

Review

Pathophysiology of ACS

**Figure 1:**
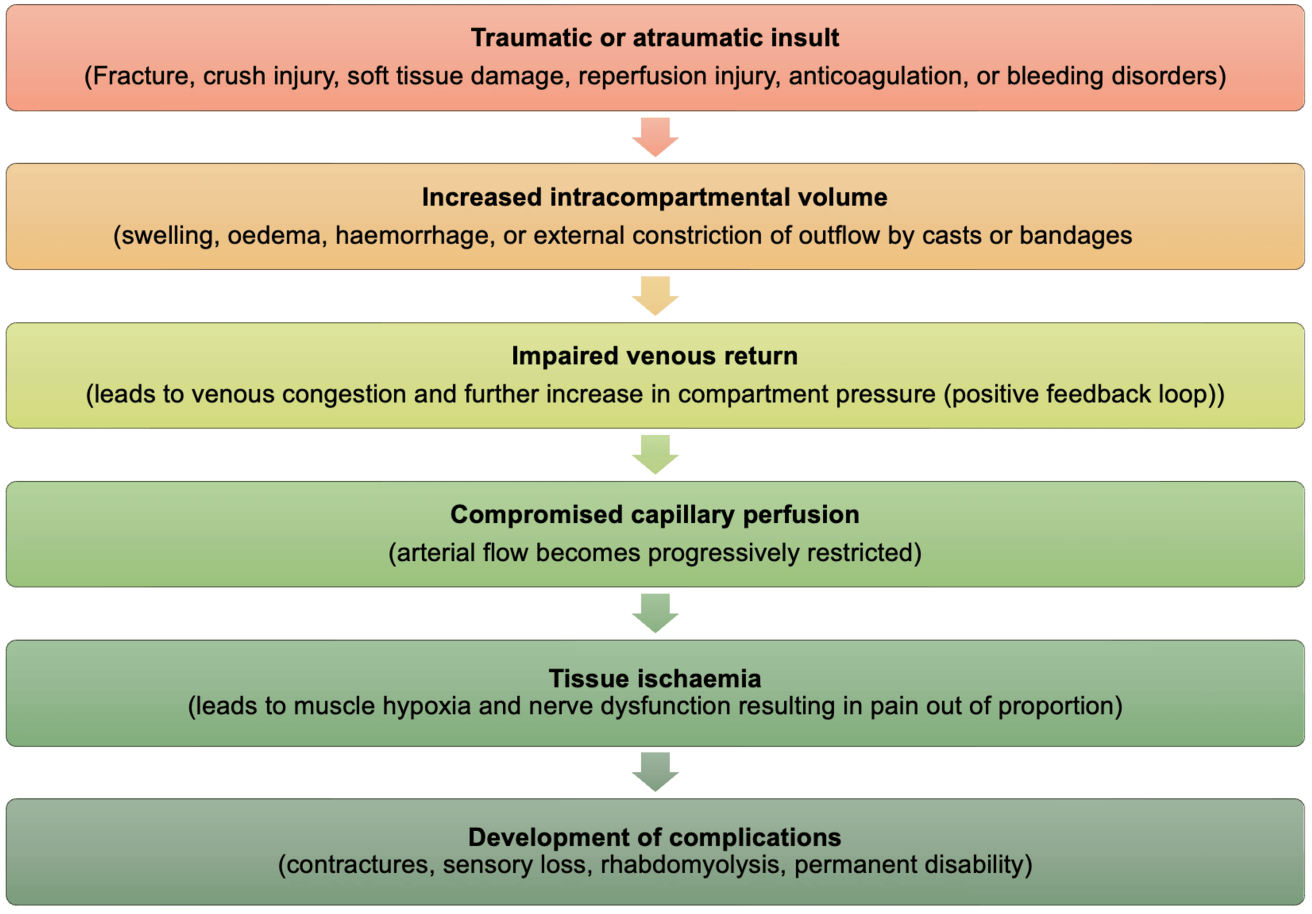
Pathophysiology of acute compartment syndrome (ACS) Image created by the author based on [3].

ACS occurs when intercompartmental pressure rises above interstitial pressure, reducing blood and oxygen supply to tissues and causing ischaemia. The rise in ICP caused vascular wall collapse as tissue pressure exceeded venous pressure. This collapse reduces tissue blood flow and, if left unchecked, leads to hypoxia. Oedema often results from tissue hypoxia and, in turn, causes further increases in ICP, reinforcing the cycle [3]. This is shown in Figure 1 below, which illustrates the cascade of events.

Risk Factors for Missed ACS

Sensory changes, including pain and neuropathy, commonly occur in missed ACS diagnoses; the neuropathy can include hyperalgesia and allodynia. ACS can damage the plantar nerves and cause the extrinsic muscles to be overpowered by the intrinsic muscles, resulting in clawing of the toes [7].

It is essential to identify factors that can result in delayed or missed ACS diagnosis. This can be ACS in patients presenting without trauma or fracture, patients with altered mental states, such as intubated or comatose patients, and those who received regional anaesthesia. Patients presenting without a fracture can face diagnosis delays of up to 13 hours, as shown by some studies. It is therefore essential to ensure regular monitoring and assessment of the compartments in comatose patients and post-regional anaesthesia patients [8].

Aya et al. (2021) conducted and published a retrospective patient review from the United States National Trauma Data Bank (NTDB). They analysed a total of 184,612 patients who underwent tibia shaft fracture fixation procedures between 2007 and 2016. A total of 1269 patients out of 184,612 had a missed diagnosis of ACS. They evaluated the associated risk factors and identified a higher risk with male gender, older population, and excessive alcohol intake, as well as smoking [1].

Intraoperatively, patients can develop ACS due to their positioning, which unfortunately is not easily detected while the patient is under general anaesthesia. One of the most common positions associated with ACS development is the lithotomy position, usually used during gynaecological and urological procedures. In addition, gynaecologists and urologists are not usually involved in the management of compartment syndrome, which can result in delayed or missed diagnosis [9].

Local Musculoskeletal Complications

Muscle necrosis and fibrosis: ACS can affect a variety of body parts and result in muscle damage, reversible or irreversible, depending on the time interval between symptom onset and surgical decompression. The most common cause is long bone fractures, with the tibia most commonly involved. The timing of surgical decompression is a key factor in controlling the risk of complications. Table 1 below summarises known complications of ACS and their association with ischaemia duration.

**Table 1:** Complications of missed acute compartment syndrome (ACS) in relation to ischaemia duration Table created by the author based on [10].

Ischaemia Duration	Primary Tissue Effects	Typical Clinical Features	Reversibility
Up to 30 minutes	Early neural irritation	Paraesthesia, heightened sensitivity	Reversible
2–4 hours	Beginning muscle dysfunction	Emerging motor weakness	Reversible
More than 4 hours	Muscle necrosis and release of myoglobin	Worsening weakness, potential acute kidney injury, or tubular necrosis	Often irreversible
Over 12 hours	Extensive neuronal loss, myonecrosis		Irreversible

Unfortunately, there is a lot of controversy when it comes to the right timing for fasciotomy. Despite the existence of evidence against delaying management, as it leads to poorer outcomes, it is not yet known when the exact timing should be ideal for fasciotomy [11].

There is also controversy about the management of missed compartment syndrome. It is essential to balance the possibility of salvaging threatened but viable soft tissue with the risk of causing further insult or infection, which can later result in more drastic measures such as amputations [12].

Nerve injury: Various studies looked at the rise in ICP and how it affects the function of human nerves, with most studies using external devices and wick catheters to gauge the critical pressure thresholds resulting in nerve dysfunction. Some studies examined the compression effect on the median nerve by applying external pressure compressing the wrist to increase ICP to 40, 50, 60, and 70 mmHg and measuring nerve conduction at each of these pressures. A pressure of 40 mmHg showed altered sensation and mild weakness, whereas higher pressures, such as 50 and 60 mmHg, or higher, showed altered/absent sensation with pronounced muscle weakness for the former and absent sensation coupled with muscle paralysis for the latter [13].

Volkmann's ischaemic contractures: Richard von Volkmann (1830-1889) was a German surgeon interested in a variety of surgical areas and recognised ischaemic paralysis [14]. His paper is widely quoted to this day, and the contractures are named as Volkmann's ischaemic contractures, which are widely associated with missed compartment syndrome. These contractures are irreversible and can affect the flexor muscles of the hand due to forearm ischaemia [15]. In the forearm, during the ischaemic event, the deep flexor muscle group is the most commonly affected. This includes the flexor pollicis longus (FPL) and flexor digitorum profundus (FDP). As ischaemia duration prolongs, muscles such as the superficial wrist flexors, pronator teres, and flexor digitorum superficialis (FDS) can also be involved [15].

Volkmann's ischaemic contractures can be classified into three main categories: mild, moderate, and severe. In mild contracture, the affected area and segment are limited. The FDP is partially damaged, with a minor sensory effect. In moderate contracture (the classic type), the FDP and FPL effects are near-total, whereas the FDS is partially affected. The fingers begin to show clawing. And finally, in severe contracture, all flexors and some extensors are affected. The sensory and motor function is likely irreversibly affected, and these patients have a high likelihood of limb amputation [16].

Functional Outcome and Limb Loss

Permanent disability: Multiple case reports have been published on individual patient cases, highlighting complications of delays in management. An example of this was a case report written by Jones et al. in 2021, published in the Journal of Cardiovascular Revascularization Medicine. It described the case of a 64-year-old male patient who underwent percutaneous coronary intervention (PCI) to his coronary artery. Postoperatively, the patient developed a haematoma and was serially assessed during his hospital stay; vascular advice was also taken. As the haematoma reduced in size within a 48-hour period, a computed tomography angiogram did not show any active bleeding. His main symptom was pain without any paraesthesia or effect on distal pulsations (radial and ulnar). He was discharged after six days of serial assessment and re-presented two days post-discharge with continuous pain. He was sent home by the emergency department with analgesia and elevation advice. Unfortunately, after 10 days, he presented to the emergency department with a painful, swollen, tense arm with a reduction in ulnar and median nerve sensation. Despite the assessments and clinical presentation, it was concluded that he did not have clinical evidence of compartment syndrome, and he was once again discharged, with a follow-up scan arranged. Upon subsequent review, he developed fixed flexion of his third and fourth digits in addition to loss of hand sensation. His scans showed a large pseudoaneurysm compressing the surrounding muscles and vasculature. Despite months of intensive therapy, he was unable to regain any function in his right hand [17].

If managed promptly, permanent functional disability can usually be avoided, and patients make an almost full recovery. However, delays in management can result in short- or long-term complications, including hypersensitisation, which results from injured tissue stretching. Patients usually undergo sensory re-education to aid with this [16].

Amputation: Amputation can be one of the major consequences of delayed management of ACS and, in turn, leads to long-term disability. It is therefore pertinent to ensure the timely identification of ACS and surgical decompression. The risk factors associated with higher rates of amputation post ACS diagnosis have not been accurately established; some studies identified a lack of intercompartmental pressure monitoring, diabetes mellitus, and low albumin levels as risk factors, while other studies identified smoking, open fractures, and high-level energy injury mechanisms as the predictors [18].

In 2024, Wang et al. published a paper on the predictors of amputation in patients with ACS following tibia fractures. They conducted a study on patients between January 2010 and September 2023 who were diagnosed with ACS and divided them into two main groups: amputated and non-amputated patients. This helped them analyse the predictors of amputation between the two groups. The study identified the following four independent risk factors for amputation: deep vein thrombosis (DVT), injury from heavy objects, crush injuries, and muscle necrosis [19].

In some studies, it is recommended to proceed with immediate amputation if the limb or digits are already ischaemic at the time of arrival to the emergency department. Patients arriving with such advanced stages should have their renal function monitored [8].

To date, there is no consensus on the actual predictors of amputation, and this was identified as a gap in the literature.

Infective Complications

Wound infection and osteomyelitis: The only effective management for compartment syndrome is fasciotomy of the affected compartment, which presents its own post-operative challenges, including the need to identify the correct timing for wound closure. Closing too early risks a rebuild-up of ICP, while delaying closure can lead to wound infection and a longer hospital stay. The existing literature lacks consensus on the optimal timing and method for fasciotomy wound closure, and it is mostly based on surgeon preference and soft-tissue condition [20]. Proximal tibia fractures that are complicated with ACS result in multiple surgeries and can often be complicated by osteomyelitis and longer patient rehabilitation [21].

There are many complications associated with fasciotomies, which include, and are not limited to the following: altered sensation of the associated skin segment (affects about 77% of patients), dry, scaly skin, wound discolouration, herniation of muscle, tethered scars or tendons, and chronic venous insufficiency due to impairment of calf muscle pumps [22].

Sepsis and systemic spread: Sepsis is one of the complications of missed compartment syndrome and delayed management, resulting in acute kidney injury, multi-organ failure, and death in some patients. Sepsis usually results from infection of necrosed muscle and, if not thoroughly debrided, can worsen the systemic condition. It is therefore essential for trauma and orthopaedic surgeons to assess and monitor patients for signs of sepsis or acute kidney injury resulting from the evolving necrosis within limb compartments [9].

Systematic Complications

Electrolyte imbalance, rhabdomyolysis, and acute kidney injury: Post-fasciotomy, patients experience varying degrees of pain and thus require strong analgesia on a regular basis. It is essential to ensure adequate hydration and to monitor renal function for any signs of failure. Urine output should be monitored, aiming for >0.5 mL/kg of urine output in addition to fluid administration intravenously [22].

With delays in ACS diagnosis and management, systemic complications can arise, including rhabdomyolysis, which can worsen renal function and lead to increases in creatinine kinase and serum creatinine levels [8].

Rhabdomyolysis can occur post-traumatic ACS and can lead to acute kidney injury (AKI), which in turn results in higher patient morbidity and mortality. Tsai et al. published a retrospective study in 2015, conducting a six-year retrospective chart review from January 2006 to March 2012. Out of the 52 patients identified with ACS, 23 of them had rhabdomyolysis (44.2%), out of which nine patients developed AKI with a significant correlation between AKI development and the presence of ischaemic injury [23].

Multi-organ failure and death: In delayed management of ACs, myoglobin levels can rise as a result of muscle damage, which in turn can cause AKI, acidaemia, and hyperkalaemia, which lead to cardiac arrhythmias and death [24].

Rehabilitation and Long-Term Sequelae

Post-fasciotomy, rehabilitation is a vital step to ensure restoration of extremity function. Due to the persisting oedema, it is not advisable to primarily close fasciotomy wounds during the initial procedure. Depending on the centre, closure can sometimes be aided using vacuum-assisted closure (VAC) systems [16].

Rehabilitation usually involves protection, resting, icing, compressing, and elevation, which are essential to reduce oedema and haematoma formation. The combination of these helps restore joint range of movement [16].

Medicolegal Consequences

In the majority of patients, compartment syndrome is a clinical diagnosis that requires urgent surgical fasciotomy. In some patients, acute lower limb ischaemia occurs as a sequela to atherosclerosis or trauma. The recommended 'cutoff' time for performing prophylactic fasciotomy is six hours [25].

Delays in ACS diagnosis and management are one of the leading causes of litigation against orthopaedic surgeons [1]. Trauma and orthopaedic surgery ranks amongst the top five specialties facing annual malpractice claims. In Germany, approximately 30% of claims are against trauma and orthopaedic surgeons [26]. In the United States, from 1991 to 2005, malpractice claims were evaluated, and 14% were against trauma and orthopaedics, followed by neurosurgery at 19.1% [27].

In the United Kingdom, a review of 124 individual jury verdict cases was carried out using the Westlaw database from January 2020 to January 2018. The most common complication was traumatic compartment syndrome of the lower limbs, which resulted in damaged nerves due to a failure to diagnose.

**Figure 2:**
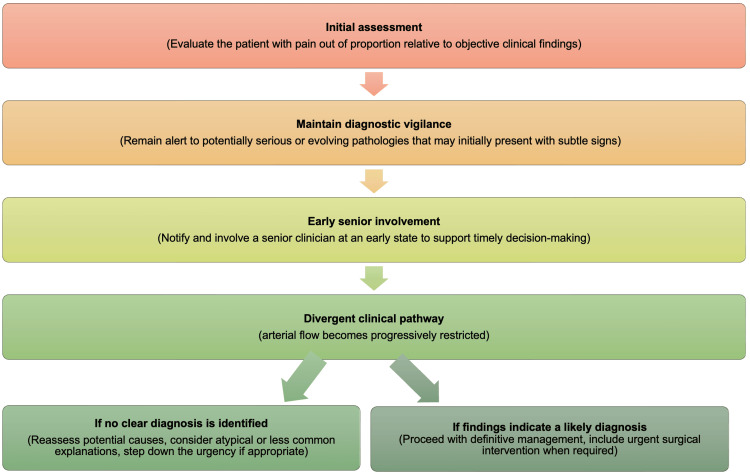
Example flowchart to aid risk reduction in patients presenting with pain out of proportion Image created by the author based on [10].

When a patient presents with pain out of proportion, local hospital guidelines need to exist to ensure the correct and timely identification of symptoms, diagnosis, and appropriate management. Figure 2 below illustrates a flowchart that summarises an appropriate response to the presentation and escalation of these patients. Of course, each hospital and Trust is likely to have their own protocols in place to ensure appropriate assessment and management.

From 2004 to 2014, the National Health Service (NHS) in the UK paid out approximately £1.5 billion in lawsuits, affecting a variety of specialties, including trauma and orthopaedic surgery, vascular surgery, and obstetrics. Between 2000 and 2006, 1473 litigation cases were due to infection, consent and mismanagement of fractures, cauda equina, and compartment syndrome [28].

In the USA, ACS indemnity payments exceed average payments ($136,000) for trauma and orthopaedic surgeon malpractice. The case settlements vary from $52,500 to $3,500,000. For cases that were not settled and resulted in court proceedings, indemnity payments ranged from $106,970 to $22,565,000. These payments varied in accordance with the level of harm affecting patients and long-term morbidities [27].

Medicolegal data, internationally, reveal that ACS is widely recognised as a high-liability condition due to the extensive consequences of delayed or missed diagnosis. In the USA, literature reviews were conducted on 358 ACS-related malpractice cases, with an average indemnity payment exceeding $3.2 million. The surgeons most frequently affected by these lawsuits were trauma and orthopaedic surgeons. There is unanimous agreement that appropriate documentation of compartment-pressure monitoring and clinical examination findings was essential, and inadequate documentation increased the risk of litigation [29], with some case settlements reaching as high as $22 million. Diagnostic delays remain as one of the strongest predictors of adverse legal outcomes [27].

Across Europe, comparable trends underscore the importance of appropriate documentation and the avoidance of diagnostic delays. These international findings highlight the importance of early recognition and the use of structured national or local protocols, such as serial neuromuscular assessments or observation charts specific to ACS, and, in countries like the UK, adherence to the BOAST guidelines [30].

## Conclusions

ACS is one of the most common surgical emergencies in trauma and orthopaedics and, in turn, is also a common cause of litigation due to delays in management or missed diagnosis in certain patients. Obtunded patients, regional block patients, and those undergoing prolonged surgery under general anaesthesia are often at a higher risk of developing compartment syndrome, which can be missed in the absence of clear communication from such patient cohorts.

The literature is extensive in identifying the signs and symptoms of compartment syndrome and the diagnostic methods, which include clinical assessment and the use of ICP in certain patients. However, the literature is not as extensive when describing systemic complications such as rhabdomyolysis, renal failure, multiorgan failure, and death due to a delayed or missed diagnosis of ACS.
